# Bilateral cochlear implantation in a child with Waardenburg syndrome: A case report

**DOI:** 10.3389/fped.2022.965884

**Published:** 2022-09-12

**Authors:** Xiaohui Wang, Lin Xu, Na Zhang, Ying Zhao

**Affiliations:** ^1^Department of Anesthesiology, Cheeloo College of Medicine, Qilu Hospital (Qingdao), Shandong University, Qingdao, China; ^2^Department of Anesthesiology, Qilu Hospital of Shandong University, Jinan, China

**Keywords:** Waardenburg syndrome, cochlear implantation, airway, anesthesia management, rare disease

## Abstract

**Background:**

Waardenburg syndrome (WS) is a rare genetic disorder that presents with clinical characteristics such as white forelock, congenital deafness, dystopia canthorum, and heterochromia iridis. It is mostly transmitted through an autosomal dominant mode, with a few genetic mutations. Children with WS often require surgical intervention at an early age and may have a difficult airway, which can be challenging for anesthesiologists.

**Case presentation:**

We report the anesthetic management during cochlear implantation in a 14-month-old girl with WS who weighed 9 kg. In addition to hearing loss and delayed speech, she presented with motor developmental delay, chewing and swallowing impairments, and dietary bucking. Resistance was encountered during tracheal intubation after anesthesia induction, and the tracheal tube was successfully intubated after rotation.

**Conclusions:**

We report the anesthetic management during cochlear implantation in a child with WS, briefly describe the research advances related to WS, and discuss the optimization of the perioperative management of these children, including airway management, anesthesia-related complications, and the use of anesthetics.

## Introduction

Waardenburg syndrome (WS) is a rare genetic disorder first described in 1951 by Waardenburg as a combination of six chief characteristics: white forelock, partial or total heterochromia iridis, congenital deafness, lateral displacement of the medial canthi and lacrimal points, high broad nasal root, and synophrys. The prevalence of WS is estimated to be 1/42,000 (1). The four subtypes of WS are defined by the presence or absence of additional symptoms: type 1 WS (WS1), dystopia canthorum; type 2 WS (WS2), no additional features; type 3 WS (WS3) or Klein–Waardenburg syndrome, symptoms of WS1 combined with musculoskeletal abnormalities of the upper limbs that often affect the choice of anesthetics; and type 4 WS (WS4) or Shah–Waardenburg syndrome, an unusual variant of WS 2 associated with Hirschsprung's disease presenting as congenital megacolon or gastrointestinal atresia. Types 1 and 2 are the most common types of this syndrome, whereas types 3 and 4 are rare (2). Six genes are currently known to be involved in this syndrome, namely, *PAX3, MITF, EDN3, EDNRB, SOX10*, and *SNAI2*, and it is likely that a number of genes remain undiscovered.

Children with WS usually require anesthesia for surgical treatment of hearing loss, Hirschsprung's disease, gastrointestinal atresia, and intestinal obstruction at an early age. However, these children often show accompanying multisystem abnormalities, and the younger the children, the greater the challenge for anesthesiologists.

Current reports of WS are primarily sporadic and do not include large samples and guidelines or expert consensus for WS, posing several challenges for anesthesia management. Therefore, we report and analyze the anesthetic management of a child with WS who underwent surgery for bilateral cochlear implantation, with the aim of providing a reference for other anesthesiologists.

A 14-month-old girl weighing 9 kg was admitted for hearing loss and language retardation. She underwent examination of otoacoustic emission at 8 months of age, but binaural hearing screening was not passed. Auditory brainstem response confirmed no response to 99 dB in either ear, and temporal bone computed tomography (CT) showed bilateral semicircular channel dysplasia. She was diagnosed with sensorineural hearing loss and was scheduled for bilateral cochlear implantation.

## Case presentation

### Pre-Anesthesia evaluation

#### Appearance

**Figure 1:**
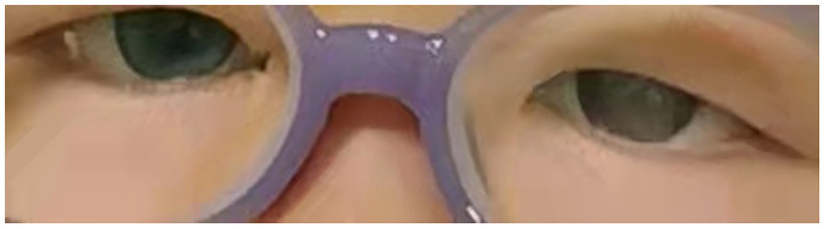
Binocular iris appearance of the patient.

The girl had a good nutrition status and a short stature. Heterochromia iridis were present in the form of a blue right eye and a brown left eye (Figure 1). In addition to hearing loss and delayed speech, the patient presented with motor developmental delay and was unable to walk or sit unaided. The girl also showed chewing and swallowing impairments to the extent that she could only consume mushy food. She often bucked while eating, and was unable to breathe under severe circumstances.

#### Supplementary examination

**Figure 2:**
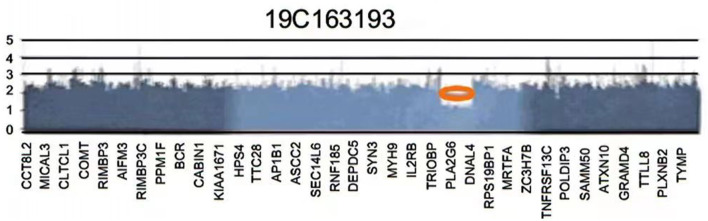
The patient's gene testing reports show a deletion of an ~1.013 Mb on the long arm of chromosome 22.

Genetic testing showed a heterozygous deletion of ~1.013 MB on the long arm of chromosome 22. Neither of her parents showed a deletion in this region, and she was an individual with mutations. The pathogenic genes in the deletion group included *DNAL4, PLA2G6*, and *SOX10* (Figure 2). Therefore, the patient was diagnosed with WS. All laboratory test results were within normal ranges. ECG showed sinus rhythm with a heart rate of 116 beats/min. Her parents were in good health and there was no history suggestive of maternal gestational disease, exposure to harmful factors during pregnancy, birth hypoxia and jaundice, drug allergy, or anesthesia-related problems.

#### Airway assessment

**Figure 3:**
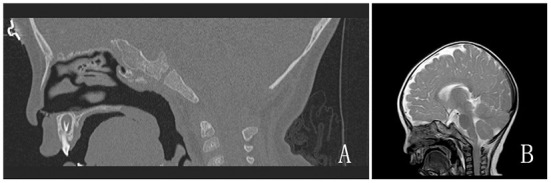
**(A,B)** Preoperative imaging examinations. **(A)** Temporal bone CT; **(B)** Brain magnetic resonance imaging.

The Mallampati score could not be evaluated in the girl because she was uncooperative. Restricted mouth opening, cervical immobility, maxillary and mandibular deformities, and other hypoplasias were not observed. Preoperative imaging showed no evidence of tracheostenosis or airway abnormalities (Figure 3). Electronic laryngoscopy and lower airway examination were not performed.

### Anesthesia process

Upon arrival to the operating room, standard monitoring was applied, peripheral venous access was established, and SpO_2_ was 100% in room air. In addition to direct laryngoscopy and endotracheal tube (ETT) with an inner diameter of 3.5 mm, we also prepared materials for a difficult airway, including video laryngoscopy, laryngeal mask airway, and fiberoptic bronchoscopy, and assigned two anesthesiologists for this patient. We intravenously administered 30 mg propofol after pre-oxygenation. Once adequate mask mechanical ventilation was performed, 1 mg of cisatracurium and 20 μg of fentanyl were administered to facilitate orotracheal intubation. Dexamethasone (2 mg) was administered to prevent airway spasms, edema, and postoperative nausea and vomiting. Endotracheal intubation was performed by the attending anesthesiologist using a direct laryngoscope. Transoral placement under direct laryngoscopic visualization revealed the epiglottis and glottis; the former appeared to be redundant and curly leaf-shaped. A reinforced ETT with a sealing cuff [internal diameter (I.D.), 3.5 mm] was chosen for intubation. However, since the forepart of the trachea passed the glottis and advanced past it, the anesthesiologist encountered some resistance and inserted the ETT by rotating it slightly. After intubation, auscultation of breath sounds was routinely performed to confirm the location of the tube, which was fixed at a depth of 13 cm from the upper incisors and connected to a mechanical ventilator. Respiratory parameters were as follows: volume control mode; tidal volume, 70 mL; respiratory rate, 20 breaths/min; fraction of inspired oxygen, 60%; and oxygen flow, 2 L/min. Anesthesia was maintained by a combination of propofol 100 mg/h and remifentanil 0.2 μg/kg·min during the surgical procedure, and no volatile anesthetics were administered.

An otolaryngologist performed bilateral cochlear implantation with the patient in the supine position. The operation was continued for 2 h 50 min. The patient's hemodynamics and body temperature were stable throughout the procedure. A total of 230 mL of crystalloid fluid was infused intraoperatively, and the blood loss volume was 3 mL. At the end of the surgery, propofol and remifentanil were terminated. Body movements and spontaneous breathing appeared 20 min after the operation. Subsequently, the patient was extubated. Phlegm sounds could be heard in the throat after extubation, but the patient could not swallow on her own or cough the phelgm out. Suction catheters were then used to carefully aspirate the sputum. After close observation for 20 min, we confirmed that her autonomous respiration, muscle strength, and response to stimulus were all normal, and the SpO_2_ was 100%. The child was then sent back to the ward by the surgeon. She showed no incision infection, fever, or operative and anesthesia complications, and was discharged on postoperative day 5. The patient visited the hospital for a return visit and device switch-on 1 month after the operation. She is currently undergoing speech rehabilitation, and we will continue to perform follow-up.

## Discussion

**Figure 4:**
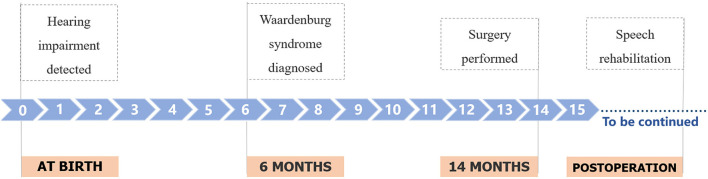
Timeline of the patient's medical history.

In this case, the baby failed the binaural hearing screening at birth and did not respond to knocks and other sounds. She was taken to several hospitals because of hearing impairment and developmental problems. When she was 3 months old, she underwent a full hearing test and was diagnosed with sensorineural deafness. Genetic testing was performed at 6 months, and the diagnosis was sensorineural deafness and WS. At 8 months age, she was taken to our hospital for the first time. Bilateral cochlear implantation was performed at 14 months of age (Figure 4).

This case met the diagnostic criteria for WS 2, considering the presence of deafness, heterochromia iridis, and the absence of dystopia canthorum and Hirschsprung disease. The patient presented with heterochromia iridis (a blue right eye and a brown left eye), which, in addition to the congenital sensorineural deafness, is associated with melanocyte depletion. In addition to the retina, melanocytes originate from neural crest (NC) cells, which further give rise to frontal bone, muscle, and intestinal ganglia. Melanocytes are not only responsible for hair, skin, and eye pigmentation but are also critical for the generation of K+ gradient between plasma membrane and intraauricular lymphatic fluid. This fluid is necessary for hair cells to transmit sound. It is also present in the stria vascularis and is necessary for normal cochlear function (3).

Patients with WS often present with a difficult airway. Both pigment-producing cells and laryngeal cartilage have been reported to have a common origin, the NC cells. Defective development of the NC results in aberrant proliferation, survival, migration, or differentiation of melanocytes and other NC-derived cells (4). Abnormal differentiation and migration of NC-derived cells may account for the laryngomalacia and epiglottis redundancy in WS. We prepared video laryngoscopy, laryngeal mask airway, and multiple types of ETTs for a possible difficult airway, and two anesthesiologists were in charge of anesthesia induction. Direct laryngoscopic visualization revealed a redundant and curly leaf-shaped epiglottis. Resistance was encountered during tracheal intubation after anesthesia induction, and the tracheal tube was successfully intubated after rotation. Numerous factors contributed to the difficult airway in this patient. Epiglottic redundancy, glottic stenosis, and compensatory hypertrophy of the lingual body induced by a long-term fluid diet may have further complicated the airway conditions. Thapa et al. reported the case of a 45-day-old infant with WS, with an omega-shaped, spongy epiglottis, similar to our 14-month-old patient, with redundant arytenoid cartilage folds and exaggerated arytenoid cartilage (5). A case of a 4-year-old child with WS was reported by Peker et al., who calculated the ETT size using the known formula (age/4+4). However, the patient could not be intubated with number 5.0 or 4.5 ETTs. Therefore, the patient was intubated with a number 3.0 uncuffed ETT (6). Michalek et al. reported the case of 46-year-old patient with WS. Owing to limited mouth opening and neck movement, direct laryngoscopy and endotracheal intubation had failed. Therefore, superior glottic airway was placed first, and endotracheal intubation was successfully performed under the guidance of a fiberbronchoscope (7).

Chewing and swallowing impairment in the 14-months-old girl in this report may be related to peripheral demyelinating neuropathy and central demyelinating leukodystrophy caused by the *SOX10* gene (8). Laboratory examinations may reveal malnutrition and electrolyte imbalance due to long-term semi-liquid or liquid diets (9). Routine blood test results and electrolyte values were within the normal range for this patient. She presented with choking on diet, an impaired pharyngeal reflex, and poor expectoration capability. Such patients are prone to airway obstruction, aspiration, and asphyxia after removal of the ETT during the anesthesia recovery period. As a result, we prepared suction equipment and materials for reintubation. The patient was extubated after sobering and regaining muscle strength. She was given oral cavity suction at the same time to keep the upper respiratory tract open and to prevent aspiration.

Two points were worth consideration with regard to anesthetics. First, muscular dysplasia was suspected since the patient was currently unable to walk or sit unaided due to delayed motor development. We used only small doses of non-depolarizing muscle relaxants during anesthesia induction, given the possibility of slowed muscle relaxant metabolism in our patient. Second, since the possibility of malignant hyperthermia associated with the gene mutations and abnormal motor development could not be ruled out, we opted to avoid the use of volatile anesthetics, and anesthesia was maintained with continuous intravenous infusion of propofol and remifentanil. Even though evidence of an association between WS and malignant hyperthermia is lacking, volatile anesthetics and muscle relaxants have been reported to be involved (10).

This study had some limitations. First, should a smaller ETT be used instead when intubation resistance is encountered in the subglottis? Although the patient did not show complications after extubation, there may have been a risk of airway edema caused by ETT rotation. The limitations of preoperative imaging examinations prevented us from measuring the subglottic airway diameter; therefore, we were unable to predict the extent of airway stenosis prior to surgery. If the preoperative image scanning range of such patients can cover the subglottic area, imaging assessments may be more helpful for preoperative evaluation of anesthesia. Another common clinical manifestation associated with WS was congenital heart disease (11–13). The patient was asymptomatic for cyanosis preoperatively, and only a subjective assessment of cardiac function based on daily symptoms was performed without objective echocardiographic data.

Therefore, preoperative assessment of these patients should be comprehensive and detailed to fully understand the related organ involvement. Especially, imaging examinations should include images of the head and neck region. Images of the subglottic area can help the anesthesiologist measure the diameter of the subglottic airway to help select the most appropriate type of tracheal tube. Echocardiogram testing should be routinely included in the preoperative examinations.

## Conclusion

We present a case of WS in a child who underwent bilateral cochlear implantation and highlight the importance of complete preoperative evaluation, identification of a difficult airway, assessment of other systemic abnormalities associated with anesthetic management, preoperative preparation of the anesthesia scheme, and perioperative focus on some special symptoms. Our case report will aid other anesthesiologists in raising awareness of WS and optimizing anesthetic management in children with WS.

## Data availability statement

The original contributions presented in the study are included in the article/supplementary material, further inquiries can be directed to the corresponding author/s.

## Ethics statement

The studies involving human participants were reviewed and approved by the Medical Ethics Committee of Qilu Hospital of Shandong University (Qingdao). Written informed consent to participate in this study was provided by the participants' legal guardian/next of kin. Written informed consent was obtained from the minor(s)' legal guardian/next of kin for the publication of any potentially identifiable images or data included in this article.

## Author contributions

XW reviewed the literature and contributed to manuscript drafting. LX contributed to manuscript drafting. NZ and YZ was responsible for revision of the manuscript for important intellectual content. All authors issued final approval for the version to be submitted.

## Funding

Support for this study was provided from the Qingdao Key Health Discipline Development Fund (2019).

## Conflict of interest

The authors declare that the research was conducted in the absence of any commercial or financial relationships that could be construed as a potential conflict of interest.

## Publisher's note

All claims expressed in this article are solely those of the authors and do not necessarily represent those of their affiliated organizations, or those of the publisher, the editors and the reviewers. Any product that may be evaluated in this article, or claim that may be made by its manufacturer, is not guaranteed or endorsed by the publisher.
